# Association between response to anti-PD-1 treatment and blood soluble PD-L1 and IL-8 changes in patients with NSCLC

**DOI:** 10.1007/s12672-023-00641-2

**Published:** 2023-03-29

**Authors:** Ling Yi, Xiaojue Wang, Siyun Fu, Zhuohong Yan, Tianyu Ma, Siqi Li, Panjian Wei, Hongtao Zhang, Jinghui Wang

**Affiliations:** 1 Department of Central Laboratory, Beijing Tuberculosis and Thoracic Tumor Research Institute, Beijing Chest Hospital, Capital Medical University, Beijing, China; 2 Department of Medical Oncology, Beijing Tuberculosis and Thoracic Tumor Research Institute, Beijing Chest Hospital, Capital Medical University, Beijing, China; 3 No. 2 Department of Thoracic Surgery, Beijing Tuberculosis and Thoracic Tumor Research Institute, Beijing Chest Hospital, Capital Medical University, Beijing, China

**Keywords:** NSCLC, Anti-PD-1 immunotherapy, Functional sPD-L1, IL-8

## Abstract

In this study, we explored the dynamic changes in blood sPD-L1 and its clinical value during anti-PD-1 immunotherapy in non-small cell lung cancer (NSCLC) patients. First, we established a sandwich ELISA for functional sPD-L1 that can bind to PD-1 and has biological functions. By monitoring functional sPD-L1 in 39 NSCLC patients treated with anti-PD-1 antibodies, we found a positive correlation between baseline sPD-L1 and tissue PD-L1 (P = 0.0376, r = 0.3581), with patients with lymph node metastasis having higher sPD-L1 levels (P = 0.0037) than those without lymph node metastasis. Although baseline functional sPD-L1 and PFS did not correlate significantly in this study, changes in sPD-L1 in patients with different clinical responses showed different trends. Blood sPD-L1 increased in 93% of patients after two cycles of anti-PD-1 treatment (P = 0.0054); sPD-L1 in nonresponsive patients continued to increase (P = 0.0181), but sPD-L1 started to decline in responsive patients. Blood IL-8 levels were associated with tumor load, and when combined with IL-8, the evaluation accuracy of sPD-L1 improved to 86.4%. This study preliminarily shows that the combination of sPD-L1 and IL-8 is a convenient and effective method for monitoring and evaluating the effectiveness of anti-PD-1 immunotherapy in NSCLC patients.

**Supplementary Information:**

The online version contains supplementary material available at 10.1007/s12672-023-00641-2.

## Introduction

Lung cancer remains the leading cause of cancer-related death around the world. Despite significant improvements in treatment, the prognosis continues to be poor [1]. Platinum-based chemotherapy for advanced NSCLC can result in a median survival period of approximately 12 months [2]. In the past ten years, landmark progress in molecular targeted therapy has led to a paradigm shift; however, the clinical benefits are limited, and drug resistance inevitably develops [3–6]. Fortunately, immunotherapy has emerged as another innovative treatment for lung cancer. PD-1/PD-L1 antibodies can lead to impressive antitumor responses by releasing the PD-1/PD-L1-mediated constraint of the immune system, and this activity has therefore become a highly promising treatment strategy for most types of cancers, especially lung cancer. Although immunotherapy based on immune checkpoint blockade actually improves progression-free survival (PFS) and overall survival (OS), the response rate remains low at approximately 20% in patients with advanced NSCLC [7–11]. In parallel, there are few concomitant biomarkers that predict the efficacy of anti-PD-1 mAb therapy, but there is an urgent need for such markers [8].

Programmed death ligand 1 (PD-L1), a member of the B7 family, is a putative type I transmembrane protein of 290 amino acids consisting of an IgV-like domain, an IgC-like domain, a transmembrane domain, and a cytoplasmic tail of 30 amino acids [12]. PD-L1 is expressed on the surface of various cell types, including macrophages, dendritic cells, endothelial cells in the heart and carcinoma cells such as lung, colon, melanoma, and leukemic cells. PD-L1 can produce an immunosuppressive environment for tumor growth through its interaction with PD-1. Interaction of PD-L1 with PD-1 on activated cytotoxic T cells results in effector T-cell exhaustion [10, 13, 14]. Blocking PD-1-PD-L1 signaling can resuscitate T cells, which has been proven to be the most successful immunomodulatory therapy to date. High PD-L1 tumor tissue protein expression has been shown to be associated with high response rates to immune checkpoint inhibitor (ICI) therapy. However, evaluating PD-L1 expression in tumor tissue is challenging, including different results of PD-L1 expression according to different anti-PD-L1 antibodies [14], heterogeneity of PD-L1 expression in tumor tissues, highly dynamic changes in advanced malignancies and inconvenience of dynamic detection, and the predicted value may be controversial [15–17].

Recently, the soluble form of PD-L1 (sPD-L1) has been measured in the peripheral blood of cancer patients, and several clinical investigations have investigated the predictive, prognostic, and regulatory roles of sPD-L1 levels in various malignancies [18–25]. These studies have revealed that sPD-L1 may impair host immunity and contribute to the systemic immunosuppression that subsequently leads to cancer progression, resulting in poor clinical outcomes. Therefore, elevated levels of sPD-L1 have been shown to predict the efficacy of various treatments, such as chemotherapy, radiotherapy, targeted therapy and immunotherapy [25–39]. In lung cancer, it has been reported that sPD-L1 is an independent predictive and prognostic biomarker for NSCLC patients receiving anti-PD-1 antibodies [33, 34]. However, there are several different PD-L1 isoforms, and different detection systems may affect detection results [23]. It seems clear that accurate sPD-L1 detection will more likely reflect the features of functional sPD-L1, with greater clinical significance [18, 28].

In this study, we dynamically detected functional sPD-L1 along with a panel of cytokines in NSCLC patients treated with anti-PD-1 antibodies. We found functional sPD-L1 and IL-8 to be valuable monitoring markers for predicting and evaluating the effectiveness of immunotherapy in NSCLC patients.

## Methods

### Patients

A total of 39 patients with NSCLC who received only anti-PD-1 antibody as a first-line or second-line treatment between 2018 and 2022 at Beijing Chest Hospital were eligible for this study. The end time of follow-up was 22 May 2022. Anticoagulant plasma was collected during treatment with anti-PD-1 immunotherapy (14 patients were treated with nivolumab, 10 with sintilimab, 8 with pembrolizumab, and 7 with other anti-PD-1 mAbs) and stored at − 80 °C for analysis of blood sPD-L1 levels. The enrolled patients met the following inclusion criteria: pathologically diagnosed lung cancer; status (ECOG PS) 0 to 1. Patients with a diagnosed second tumor in the previous 10 years and with severe life-threatening illness, receiving immunosuppressive medications or with HIV infection, autoimmunity disease, active infections, history of organ allograft and viral hepatitis were excluded. This research was approved by the ethics committee of Beijing Chest Hospital (BJXKYYFADL).

### Response metrics

Tumor burden change (maximum reduction or minimum increase in index lesions) and response status as complete response (CR), partial response (PR), stable disease (SD), or progressive disease (PD) were determined by Response Evaluation Criteria in Solid Tumors RECIST1.1.

### Generation of monoclonal antibodies to PD-L1 and affinity analysis

Mouse anti-human PD-L1 monoclonal antibodies (mAbs) were established by immunizing BALB/c (female, 6w) mice with 50 μg/mouse of human PD-L1-hFc fusion protein (Sino Biological Inc.) mixed with Freund’s Complete Adjuvant (Sigma, St Louis, MO, USA), i.p. and s.c.), followed by 3 subsequent i.p. and s.c. of 50 μg/mouse of hPD-L1-hFc given every second week together with Freund’s Incomplete Adjuvant (Sigma). One month after the last immunization, the mouse was boosted by i.p. injection and euthanized (pelltobarbitalum natricum) 4 days later, and spleen cells were hybridized with SP20 myeloma cells using standard procedures. The hybridoma cells were screened and subcloned three times for binding to only human PD-L1-His (Sino Biological Inc).

The PD-L1 mAbs in the hybridoma supernatant were purified using HiTrap Protein G antibody purification columns (GE Healthcare), and the purity of the mAbs was identified by SDS‒PAGE. The affinity of the purified mAbs was detected using ForteBio Octet Red96. First, ForteBio AMC (anti-mouse IgG-Fc capture) biosensors were soaked in a PD-L1 mAb dilution (20 μg/ml) for 300 s. Then, human PD-L1-His (diluted from 100 nM to 1.78 nM) was passed across the biosensor; on and off rates were measured to yield the Kd using standard methods.

### Identification of blocked and nonblocked PD-L1 antibodies

Human PD-1-His (1 mg/ml, Sino Biological Inc) was coated onto Costar ELISA plates. Human PD-L1-Fc at a concentration of 1.0 mg/ml and PD-L1 mAbs at concentrations of 0, 0.5, 1, and 2 mg/ml were premixed and incubated at 4 °C overnight and then added to the blocked plates at 100 µl/well for 2 h at 37 °C. The bound PD-L1-Fc was detected using goat anti-human IgG-HRP antibodies (1:5000, Jackson ImmunoResearch Laboratories Inc.) for 1 h at 37 °C. Substrate reagents (BD Biosciences) were used to detect positive reactions, and then stop solution (BD Biosciences) was added. Optical density (OD) was evaluated at 450 nm with a MULTISKAN GO instrument (Thermo Scientific). The plates were washed three times with 1 × PBS containing 0.5% Tween-20 following every step.

HEK293 cells were transfected with pCMV3-huam-PD-L1-GFPSpark (Sino Biological Inc), and a sample of 1 × 10^6^ transfected cells was incubated with or without PD-L1 mAbs (1 μg/ml) for 25 min at room temperature (RT). The cells were then incubated with human PD-1-His (1 μg/ml, Sino Biological Inc) and goat anti-His-APC (BioLegend) for 25 min at RT separately. The cells were washed twice after each staining and resuspended for analysis with an LSRFortessa flow cytometer (BD Biosciences).

### Sandwich ELISA and patient blood sPD-L1 detection

A monoclonal antibody (clone 11E3) with the ability to block PD-1-PD-L1 binding was selected as the capture antibody and coated onto ELISA plates at 2 μg/ml after blocking with 5% skim milk (BD Difco). PD-L1-His (standard) diluted from 10 ng/ml to 1 pg/ml, plasma from 40 healthy donors and 40 lung cancer patients (for ELISA system evaluation), or plasma from 39 NSCLC patients treated with anti-PD-1 mAbs (80 samples total, all samples were assayed in duplicate,1:25 diluted) was added and incubated for 2 h at RT. Biotinylated, nonblocking PD-L1 mAbs (clone 2F1) were used as detectable antibodies (2 μg/ml) and incubated for 1 h at RT, followed by incubation with streptavidin- HRP (1:3000, Cell Signaling Technology) for 0.5 h.

### Measurement of cytokines by immunofluorescence microsphere technique

APC-labeled microspheres with different fluorescence intensities coated with different specific antibodies that capture the corresponding cytokines (product code: C60011, Quantobio) were incubated with plasma samples for 2 h at room temperature. Then, biotin-labeled detection antibodies that bind with pull-down cytokines were added and incubated for 1 h,  PE-labeled streptavidin (SA-PE) was added and incubated for 30 min at room temperature. The concentrations of individual cytokines, including IL-2, IL8, TNF-α and IFN-г, in the samples and standard curves were obtained based on the fluorescence intensity of the immune complex microspheres using flow cytometry (BD LSRFortessa).

### Statistical analyses

Data analyses were performed using GraphPad Prism 8.0. Receiver operating characteristic (ROC) analysis was applied to evaluate the sensitivity and specificity of the functional sPD-L1 sandwich ELISA. Differences in PFS between patients with high and low baseline functional sPD-L1 levels were compared using the log-rank test, and the correlation of baseline functional sPD-L1 and tissue PD-L1 was analyzed using Spearman’s rank correlation. The association of baseline functional sPD-L1 with stages T, N, and M was evaluated using one-way ANOVA followed by Dunn’s post hoc test. Differences in baseline functional sPD-L1 between different TNM stages and tissue types were compared using unpaired t tests. P < 0.05 was considered statistically significant.

## Results

### Characteristics of monoclonal antibodies and establishment of sandwich ELISA

**Fig. 1:**
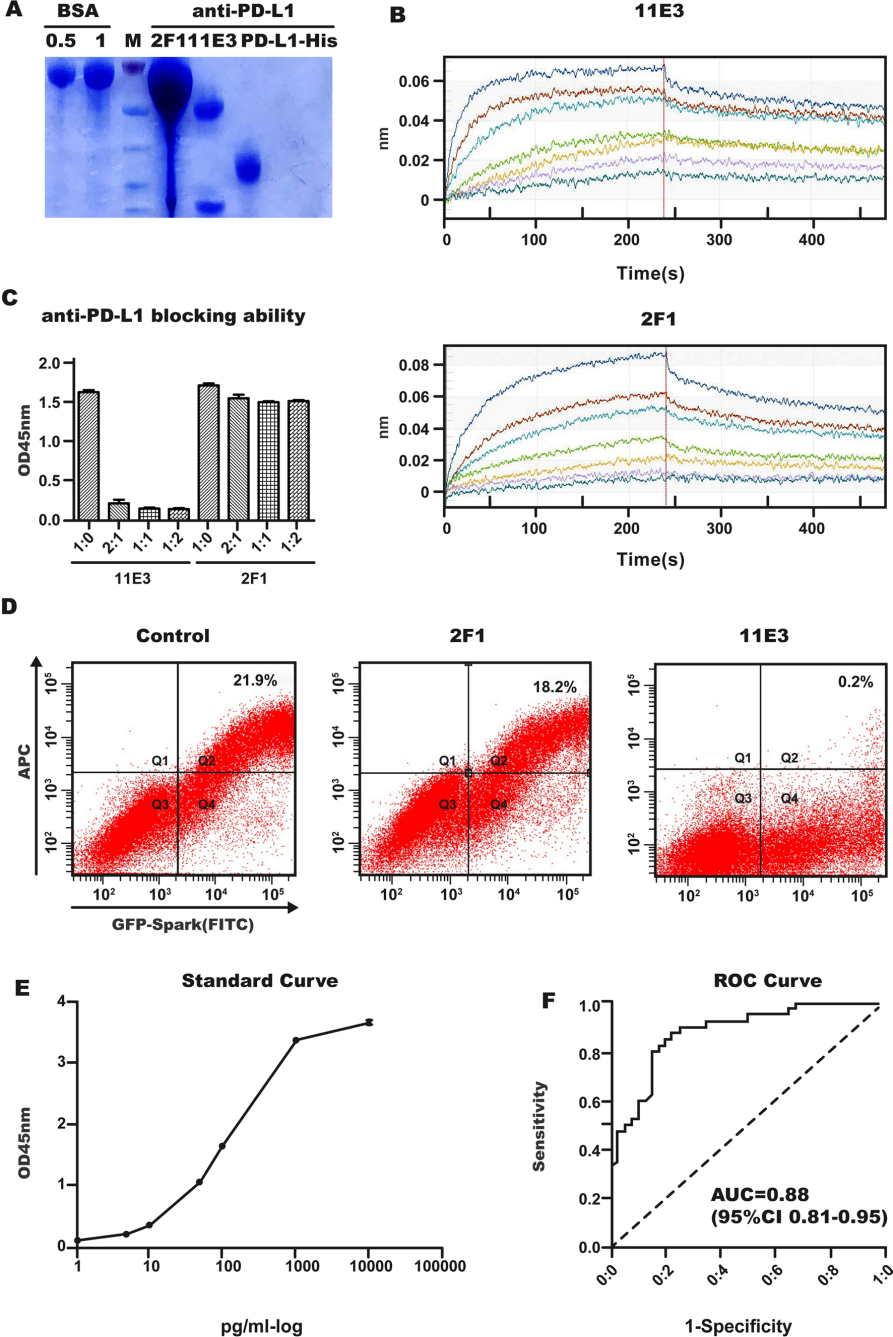
Identification of PD-L1 mAbs and sandwich ELISA. **A** mAb purity identification after affinity chromatography and SDS‒PAGE. BSA was used as the standard protein for concentration comparison. Bio2F1 and 11E3 are prepared anti-PD-L1 mAbs. And PD-L1-His is the standard for sandwich ELISA. **B** mAb binding and release curves for Kd values analyzed by ForteBio. 11E3 (upper) and 2F1 (bottom). Curves from top to bottom represent concentrations of PD-L1-His of 100, 50, 25, 12.5, 6.25, 3.13, and 1.78 nM. **C** mAb blocking ability analyzed by ELISA. 11E3 (left panel), 2F1 (right panel). **D** mAb blocking ability analyzed by flow cytometry. Left, control; middle 2F1, right, 11E3. **E** Standard curve of sandwich ELISA detecting functional sPD-L1. **F** ROC curve of sandwich ELISA. Functional sPD-L1 was obtained from 40 healthy controls and 40 lung cancer patients

A panel of over 10 clones of PD-L1 mAbs was obtained, and all of them were identified after three rounds of subcloning. These PD-L1 mAbs only bound to human PD-L1-His but not other proteins with a His-tag. Two clones, 11E3 and 2F1, were selected and purified from the hybridoma supernatant. The purity of the PD-L1 mAbs was > 90% (Fig. 1A). PD-L1 mAb affinity measurements were performed using the ForteBio Octet assay, and the affinities of both mAbs were 10^–9^ mol/L: 1.8 × 10^–9^ mol/L for 11E3 and 6.96 × 10^–9^ mol/L for 2F1 (Fig. 1B, Supplementary Table 1 and Supplementary Table 2). To capture functional sPD-L1, the blocking abilities of mAbs were identified by ELISA and flow cytometer analysis. 11E3 could completely block PD-1-PD-L1 binding, while 2F1 had no blocking ability (Fig. 1C, D). These two PD-L1 mAbs effectively established a sandwich ELISA in which 2F1 was biotin-labeled as a detection antibody, and then a biotin-avidin amplification system was introduced. The sensitivity of the established sandwich ELISA was between 0 and 5 pg/ml (Fig. 1E). The effectiveness of the sandwich ELISA was confirmed by detecting blood functional sPD-L1 in 40 lung cancer patients and 40 healthy controls (Fig. 1F).

### Association between baseline sPD-L1 and clinical characteristics

**Fig. 2:**
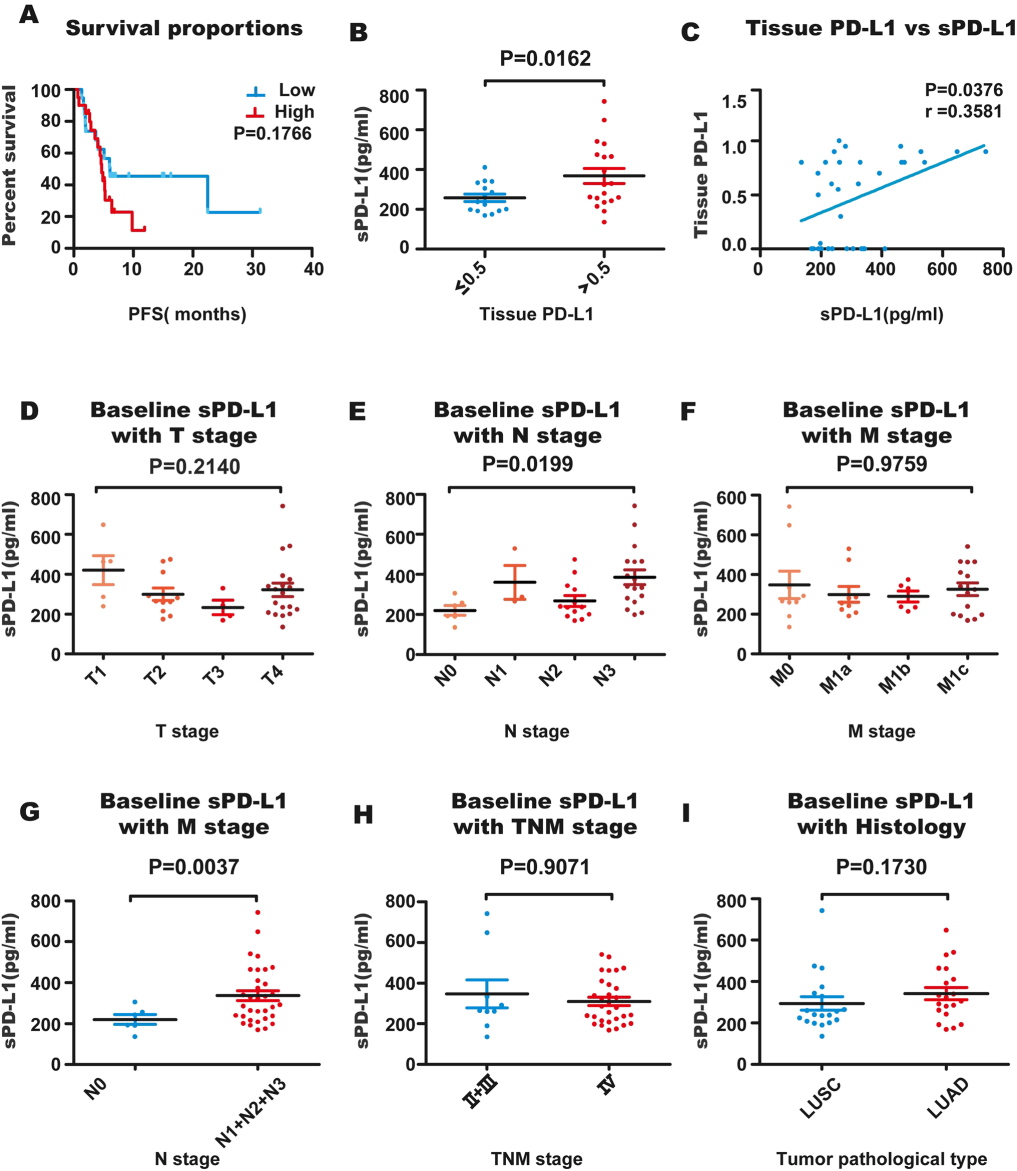
Correlation between baseline functional sPD-L1 and clinical characteristics. **A** The difference in PFS between patients with low and high functional sPD-L1 levels was compared (P = 0.1766), and dichotomization was based on the median plasma functional sPD-L1 level. **B** Baseline functional sPD-L1 was compared between patients with low and high tumor tissue PD-L1 expression (P = 0.0162). **C** Correlation between tissue PD-L1 and baseline functional sPD-L1 (P = 0.0376, r = 0.3581). **D**, **E**, **F**, Patient baseline functional sPD-L1 levels were compared among different T, N, and M stages, and there was a significant difference between the four subsets in N staging (P = 0.0199). **G** Comparison of baseline functional sPD-L1 between patients with or without lymph node metastasis (N0 vs. N1 + N2 + N3, P = 0.0037). **H** Comparison of baseline functional sPD-L1 between patients at different TNM stages (II + III vs. stage IV, P = 0.9071). Comparison of baseline functional sPD-L1 in patients with different tissue types (squamous cell carcinoma vs. adenocarcinoma, P = 0.1730)

Thirty-nine patients treated with anti-PD-1 antibodies were recruited; among them, 41% [16] were first-line treated, and 59% [23] were second-line treated. There was no significant difference in the change trend of sPD-L1 among patients groups treated with different anti-PD-1 antibodies (supplementary Fig. 1).The mean level of baseline functional sPD-L1 of the 39 patients was 318.81 pg/ml, and the median was 280.64 pg/ml. Patients with functional sPD-L1 ≥ 280.64 pg/ml tended to have shorter PFS (Fig. 2 A, P = 0.1766). The patients showed high or low tissue PD-L1 levels, and their baseline functional sPD-L1 levels were significantly different (Fig. 2B, P = 0.0162). There was a correlation between tissue PD-L1 and baseline functional sPD-L1 (Fig. 2C, P = 0.0376, Spearman, r = 0.3581). Thirty-nine patients were divided into 4 subsets according to T, N and M stage; there was no significant difference in baseline functional sPD-L1 between the four subsets (Fig. 2 D, F, P = 0.2140, P = 0.9759) in T and M staging, but there was a significant difference between the four subsets (Fig. 2E, P = 0.0199) in N staging. The baseline functional sPD-L1 of N1 + N2 + N3 patients was significantly higher than that of N0 patients (Fig. 2G, P = 0.0037). There was no significant difference in baseline functional sPD-L1 between patients with different TNM stages and different pathology types (Fig. 2H, I, P = 0.9071 and P = 0.1730). Female patients or patients without smoking had a significantly lower level of sPD-L1 before anti-PD-1 antibody therapy. The association between baseline functional sPD-L1 and clinical characteristics is summarized in Table 1.

**Table 1:** Correlation between baseline functional sPD-L1 and clinical characteristics

Characteristics	Groups	Number of patients	Mean sPD-L1 (pg/ml)	P value
Age				0.5050
	≥ 65	19	334.32	
	< 65	20	304.08	
Sex				0.0106
	Male	29	347.57	
	Female	10	235.42	
Smoking Status				0.0062
	Never	10	244.93	
	Smoker	29	344.29	
Histology				0.1730
	Adenocarcinoma	20	341.99	
	Squamous cell carcinoma	19	294.41	
PD-L1 expression				0.0162
	≤ 0.5	15	258.08	
	> 0.5	19	367.62	
Clinical T stage				0.2140
	T1 + T2	16	336.77	
	T3 + T4	23	306.32	
Clinical N stage				0.0037
	N0	6	220.52	
	N1 + N2 + N3	33	336.70	
cTNM				0.9071
	IIB-IIIC	9	347.44	
	IV	30	310.21	

### Functional sPD-L1 changes and clinical response

**Fig. 3:**
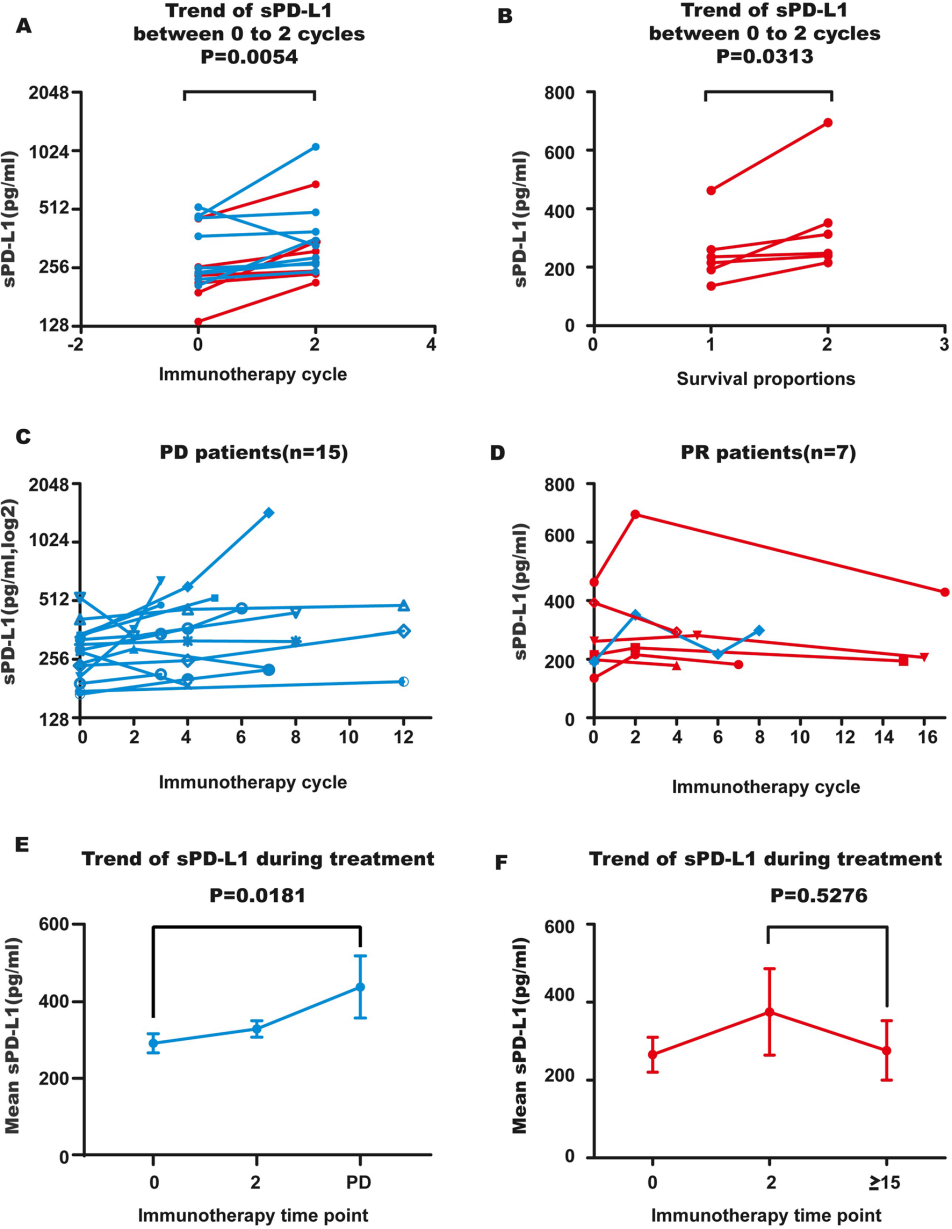
Dynamic changes in functional sPD-L1 during anti-PD-1 treatment. **A** Blood functional sPD-L1 was elevated from baseline to 2 cycles (42 days) in NSCLC patients treated with anti-PD-1 antibodies. **B** Blood functional sPD-L1 was increased from baseline to 2 cycles in PR patients. **C**, **D** Dynamic changes in blood functional sPD-L1 in PD (n = 15) and PR (n = 7) patients during anti-PD-1 treatment. Each line represents a patient. **E, F** Dynamic changes in the mean value of functional sPD-L1 during anti-PD-1 treatment. Functional sPD-L1 was gradually increased in PD patients; in the PR group, sPD-L1 rose first and then declined after 2 cycles of treatment. PD, blue; PR, red. There was one patient in the PR subset (blue line) whose sPD-L1 was increased after 8 cycles of anti-PD-1 treatment (evaluated as PR), and her treatment was changed to anti-PD-1 mAb + bevacizumab at the 19th cycle because of an increase in pleural effusion. The increase in sPD-L1 occurred earlier than did clinical symptoms

Among 39 patients, dynamic detection of functional sPD-L1 during treatment occurred in 22, and they were categorized into the PD or PR group according to whether they had PD at the time of blood collection. Among them, 15 patients with disease progression were classified into the PD group, and seven patients who did not have progression were classified into the PR group, including 2 patients in whom progression occurred later. The response was evaluated as PR during blood detection. Fifteen patients showed sPD-L1 detection at both baseline and after two cycles of immunotherapy. The results indicated that after two cycles of treatment, functional sPD-L1 increased (Fig. 3A, P = 0.0054). In the PD group, sPD-L1 continued to increase in 80% (12/15) of patients (Supplementary Fig. 2 and Fig. 3C). In the PR group, following two cycles of increase (Fig. 3B), functional sPD-L1 started to decline in 100% (7/7) of patients (Fig. 3D). Average sPD-L1 levels in patients at three representative time points were calculated, and dynamic changes in sPD-L1 levels in the PD group and PR group differed (Fig. 3E, F and Table 2). In addition, there was one patient in the PR subset whose functional sPD-L1 level increased just after two cycles of treatment and then decreased; following 8 cycles of treatment (evaluated as PR), her sPD-L1 level increased again. Her treatment was changed to anti-PD-1 mAb + bevacizumab at 19 cycles after an increase in pleural effusion, and the increase in functional sPD-L1 occurred earlier than the clinical symptoms (Fig. 3D blue line).

**Table 2:** Median values and changes in functional sPDL1 in PD and PR groups during anti-PD-1 treatment

Response	Treatment cycle		Change (pg/ml)	Treatment time point	Change (pg/ml)	
					To cycle 0	To cycle 2
	0	2 (n = paired sample)		≥ 15 (n = paired sample)		
PR	265.13 (n = 7)	374.94(n = 4)	109	275.99(n = 3)	10	− 99
				Time of PD		
PD	291.76(n = 15)	329.24(n = 3)	38	454.58(n = 15)	163	125

### Association between cytokine changes and clinical response

**Fig. 4:**
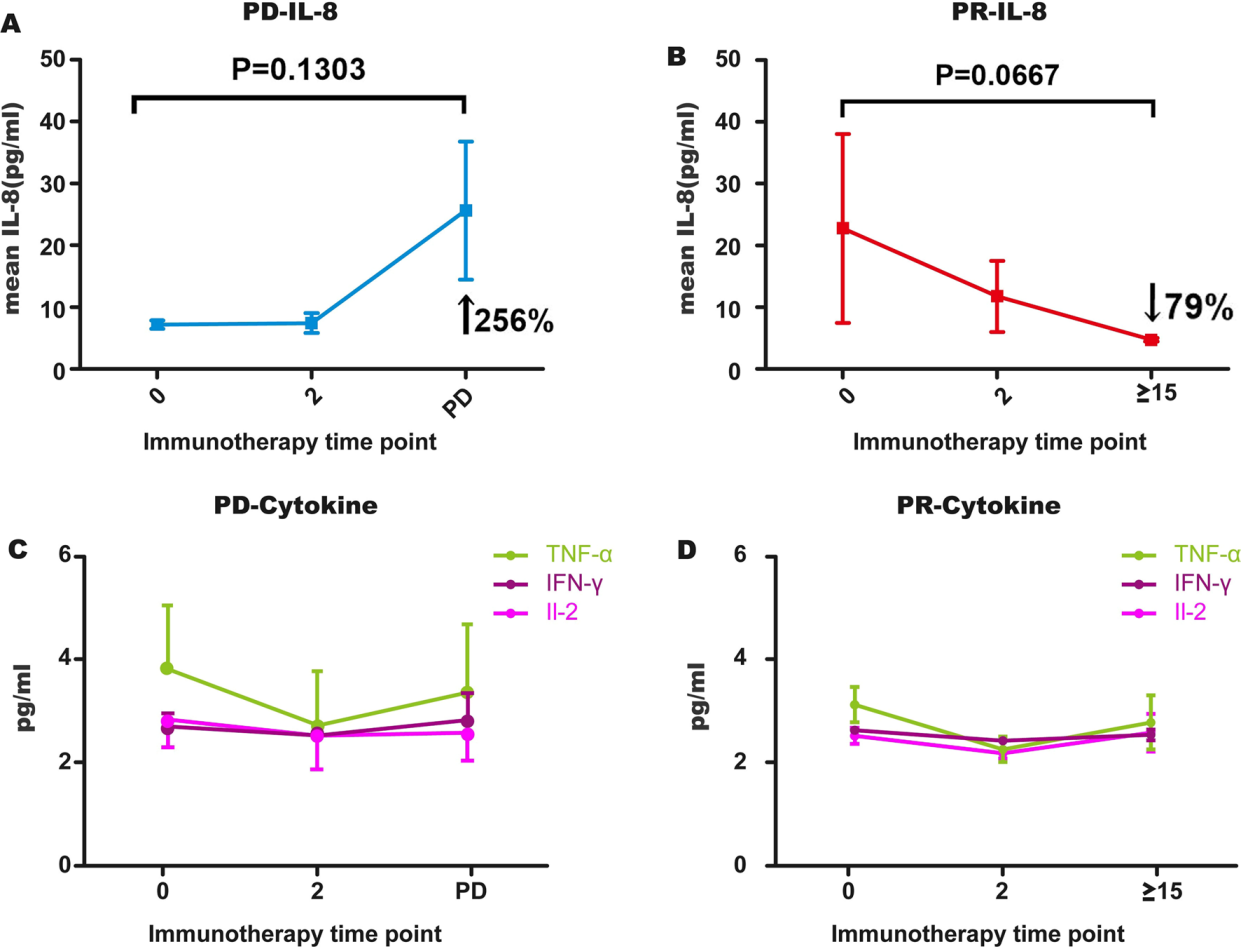
Dynamic changes in cytokines during anti-PD-1 treatment. **A**, **B** Blood concentrations of IL8 were measured during anti-PD-1 treatment at baseline, at 2 cycles of treatment and at the time of progressive disease or more than 15 cycles of treatment. Lines show the trend of the Il-8 median value at each moment **C**, **D**, Changes in mean values of IL-2, TNF-α and IFN-г in PD and PR patients during anti-PD-1 treatment. All cytokines declined from baseline to 2 cycles and then rose slightly

Dynamic changes in cytokines were also detected in the same samples with functional sPD-L1 detection. The results showed that dynamic changes in IL-8 were significantly different in the PD and PR groups (Supplementary Fig. 3). The mean level of IL-8 showed a continuous upward trend from baseline to two cycles of treatment and then reached the highest level at the progression point. In contrast, the mean level of IL-8 showed a gradual downward trend from baseline (Fig. 4A, B). IL-2, TNF-α and IFN-г were all decreased after 2 cycles of treatment and then rose slightly after 2 cycles in both the PD and PR groups (Fig. 4C, D).

### Combined detection of sPD-L1 and IL-8 for monitoring the anti-PD-1 response

Through dynamic monitoring of functional sPD-L1 and cytokines in NSCLC patients treated with anti-PD-1 mAbs, it was found that the dynamic changes in sPD-L1 and IL-8 in PD patients were different from those in PR patients; both changes were consistent with tumor load. We retrospectively analyzed the evaluation accuracy of individual sPD-L1 and combined sPD-L1 + IL-8. Functional sPD-L1 in most PD patients rose from baseline to 2 cycles and then continued to rise, reaching the highest level at the time of progression. Eleven of 15 PD cases followed this trend, and the accuracy was 73%. In addition, IL-8 levels in 8 of 15 PD patients showed a continuous upward trend from baseline to two cycles of treatment and then reached the highest level at the progression point; the evaluation accuracy was 53%. When sPD-L1 and IL-8 were combined, 12 PD patients exhibited gradually elevated sPD-L1 or IL-8 levels, and the accuracy reached 80%. In contrast, the sPD-L1 level of PR patients increased after 2 cycles of treatment and then decreased gradually, with all PR cases following this trend. The accuracy of sPD-L1 evaluation was 100%. The mean level of IL-8 gradually decreased in 5 PR patients, with 71% accuracy. The evaluation accuracy of a single marker and combined markers is summarized in Table 3.

**Table 3:** Accuracy of functional sPD-L1 and IL-8 in efficacy evaluation during anti-PD-1 treatment

Response	sPD-L1		IL-8		sPD-L1 + IL-8	
	Consistency	Accuracy (%)	Consistency	Accuracy (%)	Consistency	Accuracy (%)
PD	11/15	73	8/15	53	12/15	80
PR	7/7	100	5/7	71	7/7	100
Total	18/22	82	13/22	59	19/22	86

## Discussion

Recently, several PD-L1 splicing variants have been identified. Clinically, individual patients might express different sPD-L1 isoforms or mRNA variants. These essential studies, such as that by Bo Gong et al., have elucidated five PD-L1 splicing variants through RNA-seq to date [12]. Another noteworthy report identified four sPD-L1 variants in melanoma cell lines, and when they used two paired PD-L1 mAbs to detect blood sPD-L1 levels from the same group of cancer patients, they obtained distinct results [28]. Current commercial antibodies or ELISA kits cannot evaluate the quality and functional features of sPD-L1 [18, 28]. In view of this, we first developed paired PD-L1 mAbs targeting different binding epitopes of the extracellular domain of PD-L1. The capture mAb-11E3 clearly performed as a blocking mAb that completely blocked PD-1 and PD-L1 binding, as also confirmed at the cellular level [40]. The detection mAb was nonblocking, and the sandwich ELISA established could detect and quantify functional sPD-L1 and had the same level of sensitivity and specificity as commercial kit [41]. Functional sPD-L1 not only induces lymphocyte exhaustion and benefits tumor evasion but also competes for membrane PD-1 binding sites available to anti-PD-1 mAbs. Therefore, establishment of an effective assay for functional sPD-L1 is necessary and lays a foundation for evaluating functional sPD-L as a monitoring marker during clinical immunotherapy.

Since expression of PD-L1 in tumor tissues can effectively predict patient prognosis in some types of tumors, it is the most significant therapeutic effect prediction marker for anti-PD-1 treatment of lung cancer. However, soluble PD-L1 potentially derives from PD-L1-expressing cells, including tumor cells and immune cells. In this study, we first evaluated the correlation between the level of sPD-L1 and the prognosis of NSCLC patients treated with anti-PD-1 mAbs. It was previously reported that sPD-L1 levels and prognoses vary in patients with different tumor types receiving anti-PD-1 treatment. In patients with RCC, high baseline sPD-L1 tended to be associated with PD on nivolumab therapy, but in melanoma, high baseline levels of sPD-L1 were not associated with clinical outcomes [39]. An earlier study enrolled 233 advanced NSCLC patients treated with anti-PD-1 antibodies and found that serum sPD-L1 was an independent negative predictor of prognosis [33]. Mazzaschi G also reported longer PFS and OS in NSCLC patients with lower sPD-L1 [34]. Another report showed no difference in survival outcomes between patients with low and high sPD-L1 [42]. In our study, patients with high baseline functional sPD-L1 tended to have shorter PFS, but the difference did not reach statistical significance. However, we found a significant correlation between baseline functional sPD-L1 and tissue PD-L1 in 39 NSCLC patients, which was consistent with Shuji Murakami’s results [33]. Studies have shown sPD-L1 expression independent of tumor PD-L1 expression in some types of cancer, such as lymphoma and pancreatic cancer [39, 43–45]. As the baseline functional sPD-L1 levels of patients with or without lymph node metastasis were significantly different, functional sPD-L1 might be related to tumor invasiveness. Other studies have also reported a higher serum sPD-L1 concentration in patients with metastasis [29, 31, 33, 34, 37], suggesting that a high level of sPD-L1 is beneficial for tumor escape from immune surveillance [15]. Interestingly, we observed that female patients or patients without smoking had a significantly lower level of functional sPD-L1 before ICI therapy. Angelo Castello’s study also found that females had lower sPD-L1 and hypothesized that sex hormones regulate expression and function of PD-1/PD-L1 [42]. Nevertheless, other studies have not found this trend [19, 20, 26, 34]. In summary, the baseline level of functional sPD-L1 did not appear to show a dramatic prognostic correlation in our study but may reflect tumor load and potentially be used for monitoring therapeutic effect.

Thus far, few studies have reported dynamic changes in sPD-L1 during immunotherapy in NSCLC. In this study, we focused on monitoring changes in functional sPD-L1 during immunotherapy. Ninety-three percent of patients showed an increase in sPD-L1 after 2 cycles (approximately 42 days) of anti-PD-1 mAbs (P = 0.0054). For PR patients who benefited from anti-PD-1 treatment, the level of sPD-L1 began to decline gradually after 2 cycles of treatment (7/7), though the level of sPD-L1 in PD patients maintained a rapid increase (P = 0.0181). The 2-cycle increase in sPD-L1 concentration during ICI treatment may reflect expansion of tumor volume or tumor lysis; we believe that the former was the main reason for this in PD patients and that the latter was the main reason in PR patients. These results suggest that anti-PD-1 therapy has a turning point at 2 cycles in lung cancer and that sPD-L1 can be used as an effective monitoring marker. Recently, Mahoney KM’s research found that an increase in sPD-L1 is associated with progression in both RCC and melanoma after ICI treatment [39].

A panel of cytokines was also monitored during immunotherapy. IL-2, TNF-α, and IFN-г are related to T-cell activation or antitumor immunity, and we monitored these cytokines to determine immune status after treatment. Between baseline and 2 cycles of treatment, IL-2, IFN-γ and TNF-α decreased, which might be caused by the 2-cycle sPD-L1 increase. After 2 cycles of anti-PD-1 treatment, these cytokines began to rise slightly, reflecting activation of the immune system. The level of IL-8 in patients in whom anti-PD-1 treatment was effective showed a gradual downward trend from baseline; in patients with PD, IL-8 showed a continuous upward trend after treatment. This result is consistent with the study of Sanmamed MF and Juliá EP’s, which proves that the concentration of IL-8 in serum actually reflects tumor burden [46–48].

Since both sPD-L1 and IL-8 can reflect tumor burden, the combination of sPD-L1 and IL-8 may increase the accuracy of the evaluation of response to immunotherapy in NSCLC. We retrospectively analyzed the performance of these two markers in differentiating patients who did or did not benefit from ICI treatment. The accuracy of sPD-L1 was 82% (PD: 73%, PR: 100%), that of IL-8 was 59% (PD: 33%, PR: 71%), and that of sPD-L1 + IL-8 was 86.4% (PD: 80%, PR: 100%). If levels of sPD-L1 and IL-8 in patients increase after 2 cycles of anti-PD-1 treatment, they cannot benefit from this therapy. If sPD-L1 and IL-8 both decrease after 2 treatment cycles, patients can benefit from anti-PD-1 treatment. During anti-PD-1 treatment, monitoring sPD-L1 and IL-8 can improve the accuracy of response evaluation. Noninvasive biomarkers in blood have the advantage that measurement can be repeated and sequentially determined and thus possibly reflect changes in cancer progression during anti-PD-1 treatment. This approach may be useful in directing patient therapy in addition to pretreatment biopsy and imaging examination [49].

However, several limitations of our study should be considered. First, the sample sizes were small because there were relatively few patients receiving simple immunotherapy in our hospital, which may affect the analysis of the relationship between baseline functional sPD-L1 levels and the prognosis of patients to a certain extent. Second, the time of dynamic detection of sPD-L1 levels should be unified, and blood sPD-L1 should also be detected earlier. Third, the monitoring value of these two markers needs further clinical investigation.

Conclusion: Monitoring functional sPD-L1 during ICI treatment is valuable because patients who do or do not benefit from anti-PD-1 treatment show different sPD-L1 change trends. IL-8 monitoring during ICI treatment can improve the accuracy of response evaluation.

## Supplementary Information

Additional file1 (DOCX 12 KB)

Additional file2 (DOCX 12 KB)

Additional file 3: Figure 1 Comparison of sPD-L1 change trend among groups treated with different PD-1 antibodies during treatment. A, There was no significant difference in the change trend of sPD-L1 among different groups between 0 to 2 cycles. B, There was no significant difference in the change trend of sPD-L1 among different groups between 0 to PD or ≥15 cycles.

Additional file 4: Figure 2 Changes in functional sPD-L1 from baseline to 2 cycles in PD patients Blood functional sPD-L1 levels rose from baseline to 2 cycles in PD patients.

Additional file 5: (JPG 247 KB): Figure 3 Dynamic changes in IL-8 during anti-PD-1 treatment. A,B, Dynamic changes in blood IL-8 in PD (n=15) and PR (n=7) patients during anti-PD-1 treatment. Each line represents a patient.

## Data Availability

The datasets used and/or analyzed during the current study are available from the corresponding authors on reasonable request.

## References

[1] Torre LA, Bray F, Siegel RL, Ferlay J, Lortet-Tieulent J, Jemal A (2015). Global cancer statistics, 2012. CA Cancer J Clin.

[2] Schiller JH, Harrington D, Belani CP (2002). Comparison of four chemotherapy regimens for advanced non-small-cell lung cancer. N Engl J Med.

[3] Kris MG, Johnson BE, Berry LD (2014). Using multiplexed assays of oncogenic drivers in lung cancers to select targeted drugs. JAMA.

[4] Lin JJ, Shaw AT (2016). Resisting resistance: targeted therapies in lung cancer. Trends Cancer.

[5] Mok TS, Wu YL, Thongprasert S (2009). Gefifitinib or carboplatin- paclitaxel in pulmonary adenocarcinoma. N Engl J Med.

[6] Solomon BJ, Mok T, Kim DW (2014). First-line crizotinib versus chemotherapy in ALK-positive lung cancer. N Engl J Med.

[7] Meng X, Liu Y, Zhang J, Teng F, Xing L, Yu J (2017). PD-1/PD-L1 checkpoint blockades in non-small cell lung cancer: new development and challenges. Cancer Lett.

[8] Darvin P, Toor SM, Sasidharan Nair V, Elkord E (2018). Immune checkpoint inhibitors: recent progress and potential biomarkers. Exp Mol Med.

[9] Sznol M, Chen L (2013). Antagonist antibodies to PD-1 and B7–H1 (PD-L1) in the treatment of advanced human cancer. Clin Cancer Res.

[10] Gettinger SN, Horn L, Gandhi L (2015). Overall survival and long-term safety of nivolumab (anti-programmed death 1 antibody, BMS-936558, ONO-4538) in patients with previously treated advanced non-small-cell lung cancer. J Clin ncol.

[11] Wolchok JD, Chiarion-Sileni V, Gonzalez R (2017). Overall survival with ombined nivolumab and ipilimumab in advanced melanoma. N Engl J Med.

[12] Gong B, Kiyotani K, Sakata S, Nagano S, Kumehara S, Baba S (2019). Secreted PD-L1 variants mediate resistance to PD-L1 blockade therapy in non–small cell lung cancer. J Exp Med.

[13] Shi L, Chen S, Yang L, Li Y (2013). The role of PD-1 and PD-L1 in T-cell immune suppression in patients with hematological malignancies. J Hematol Oncol.

[14] Yu H, Boyle TA, Zhou C, Rimm DL, Hirsch FR (2016). PD-L1 expression in lung cancer. J Thorac Oncol.

[15] McLaughlin J, Han G, Schalper KA, Carvajal-Hausdorf D, Pelekanou V, Rehman J, Velcheti V, Herbst R, LoRusso P, Rimm DL (2016). Quantitative assessment of the heterogeneity of PD-L1 expression in non-small-cell lung cancer. JAMA Oncol.

[16] Taube JM, Klein A, Brahmer JR, Xu H, Pan X, Kim JH (2014). Association of PD-1, PD-1 ligands, and other features of the tumor immune microenvironment with response to anti-PD-1 therapy. Clin Cancer Res.

[17] Rasmussen JH, Lelkaitis G, Håkansson K, Vogelius IR, Johannesen HH, Fischer BM, Bentzen SM, Specht L, Kristensen CA, von Buchwald C, Wessel I, Friborg J (2019). Intratumor heterogeneity of PD-L1 expression in head and neck squamous cell carcinoma. Br J Cancer.

[18] Takeuchi M, Doi T, Obayashi K, Hirai A (2018). Soluble PD-L1 with PD-1-binding capacity exists in the plasma of patients with non-small celllung cancer. Immunol Lett.

[19] Finkelmeier F, Canli O, Tal A, Pleli T, Trojan J, Schmidt M (2016). High levels of the soluble programmed death-ligand (sPD-L1) identify hepatocellular carcinoma patients with a poor prognosis. Eur J Cancer.

[20] Okuma Y, Hosomi Y, Nakahara Y, Watanabe K, Sagawa Y, Homma S (2017). High plasma levels of soluble programmed cell death ligand 1 are prognostic for reduced survival in advanced lung cancer. Lung Cancer.

[21] Chang B, Huang T, Wei H, Shen L, Zhu D, He W (2019). The correlation and prognostic value of serum levels of soluble programmed death protein 1 (sPD-1) and soluble programmed death-ligand 1 (sPD-L1) in patients with hepatocellular carcinoma. Cancer Immunol Immunother.

[22] Shigemori T, Toiyama Y, Okugawa Y, Yamamoto A, Yin C, Narumi A (2019). Soluble PD-L1 expression in circulation as a predictive marker for recurrence and prognosis in gastric cancer: direct comparison of the clinical burden between tissue and serum PD-L1 expression. Ann Surg Oncol.

[23] Zhou J, Mahoney KM, Giobbie-Hurder A, Zhao F, Lee S, Liao X (2017). Soluble PD-L1 as a biomarker in malignant melanoma treated with checkpoint blockade. Cancer Immunol Res.

[24] Frigola X, Inman BA, Lohse CM, Krco CJ, Cheville JC, Thompson RH (2011). Identifification of a soluble form of B7–H1 that retains immunosuppressive activity and is associated with aggressive renal cell carcinoma. Clin Cancer Res.

[25] Ugurel S, Schadendorf D, Horny K, Sucker A, Schramm S, Utikal J (2020). Elevated baseline serum PD-1 or PD-L1 predicts poor outcome of PD-1 inhibition therapy in metastatic melanoma. Ann Oncol.

[26] Okuma Y, Wakui H, Utsumi H, Sagawa Y, Hosomi Y, Kuwano K (2018). Soluble programmed cell death ligand 1 as a novel biomarker for nivolumab therapy for non-small-cell lung cancer. Clin Lung Cancer.

[27] Bonomi M, Ahmed T, Addo S, Kooshki M, Palmieri D, Levine BJ (2019). Circulating immune biomarkers as predictors of the response to pembrolizumab and weekly low dose carboplatin and paclitaxel in NSCLC and poor PS: an interim analysis. Oncol Lett.

[28] Abu Hejleh T, Furqan M, Ballas Z, Clamon G (2019). The clinical significance of soluble PD-1 and PD-L1 in lung cancer. Crit Rev Oncol Hematol.

[29] Kim HJ, Park S, Kim K-J, Seong J (2018). Clinical signifificance of soluble programmed cell death ligand-1 (sPD-L1) in hepatocellular carcinoma patients treated with radiotherapy. Radiother Oncol J Eur Soc Ther Radiol Oncol.

[30] Ha H, Bang J-H, Nam A-R, Park J-E, Jin MH, Bang Y-J (2019). Dynamics of soluble programmed death-ligand 1 (sPDL1) during chemotherapy and its prognostic implications in cancer patients: biomarker development in immuno-oncology. Cancer Res Treat.

[31] Fukuda T, Kamai T, Masuda A, Nukui A, Abe H, Arai K, Yoshida K (2016). Higher preoperative serum levels of PD-L1 and B7–H4 are associated with invasive and metastatic potential and predictable for poor response to VEGF-targeted therapy and unfavorable prognosis of renal cell carcinoma. Cancer Med.

[32] Park W, Bang J-H, Nam A-R, Park JE, Kim MH, Oh KS (2019). Prognostic value of serum soluble programmed death-ligand 1 (sPDL1) and dynamics during chemotherapy in advanced gastric cancer patients. J Clin Oncol.

[33] Murakami S, Shibaki R, Matsumoto Y, Yoshida T, Goto Y, Kanda S, Horinouchi H, Fujiwara Y, Yamamoto N, Ohe Y (2020). Association between serum level soluble programmed cell death ligand 1 and prognosis in patients with non-small cell lung cancer treated with anti-PD-1 antibody. Thorac Cancer.

[34] Mazzaschi G, Minari R, Zecca A, Cavazzoni A, Ferri V, Mori C, Squadrilli A, Bordi P, Buti S, Bersanelli M, Leonetti A, Cosenza A, Ferri L, Rapacchi E, Missale G, Petronini PG, Quaini F, Tiseo M (2020). Soluble PD-L1 and circulating CD8+PD-1+ and NK cells enclose a prognostic and predictive immune effector score in immunotherapy treated NSCLC patients. Lung Cancer.

[35] Buder-Bakhaya K, Hassel JC (2018). Biomarkers for clinical benefit of immune checkpoint inhibitor treatment-a review from the melanoma perspective and beyond. Front Immunol.

[36] Han B, Dong L, Zhou J, Yang Y, Guo J, Xuan Q, Gao K, Xu Z, Lei W, Wang J, Zhang Q (2021). The clinical implication of soluble PD-L1 (sPD-L1) in patients with breast cancer and its biological function in regulating the function of T lymphocyte. Cancer Immunol Immunother.

[37] Takahashi N, Iwasa S, Sasaki Y, Shoji H, Honma Y, Takashima A, Okita NT, Kato K, Hamaguchi T, Yamada Y (2016). Serum levels of soluble programmed cell death ligand 1 as a prognostic factor on the first-line treatment of metastatic or recurrent gastric cancer. J Cancer Res Clin Oncol.

[38] Ando K, Hamada K, Watanabe M, Ohkuma R, Shida M, Onoue R, Kubota Y, Matsui H, Ishiguro T, Hirasawa Y, Ariizumi H, Tsurutani J, Yoshimura K, Tsunoda T, Kobayashi S, Wada S (2019). Plasma levels of soluble PD-L1 correlate with tumor regression in patients with lung and gastric cancer treated with immune checkpoint inhibitors. Anticancer Res.

[39] Mahoney KM, Ross-Macdonald P, Yuan L, Song L, Veras E, Wind-Rotolo M, McDermott DF, Stephen Hodi F, Choueiri TK, Freeman GJ (2022). Soluble PD-L1 as an early marker of progressive disease on nivolumab. J Immunother Cancer.

[40] Shuping L, Xiaojue W, Bin Y, Helin W, Zhuohong Y, Ling Y, Panjian W, Xin J, Jingqing H, Hongtao Zh (2020). Construcion and expression of chimeric antigen receptors targetingepidermal growth factor receptor(EGFR) and programmed cell death ligand-1(PD-L1). Chin J Microbiol Immunol.

[41] Li S, Yi L, Wei X, Zhang J, Wang X, Jiang C, Yan Z, Song L, Yang B, Wei P, Gao X, Wang J, Zhang H, Zhang J (2022). Soluble programmed cell death-ligand 1 as a new potential biomarker associated with acute coronary syndrome. Front Cardiovasc Med.

[42] Castello A, Rossi S, Toschi L, Mansi L, Lopci E (2020). Soluble PD-L1 in NSCLC patients treated with checkpoint inhibitors and its correlation with metabolic parameters. Cancers.

[43] Rossille D, Gressier M, Damotte D, Maucort-Boulch D, Pangault C, Semana G, Le Gouill S, Haioun C, Tarte K, Lamy T, Milpied N, Fest T, Groupe Ouest-Est des Leucémies et Autres Maladies du Sang, Groupe Ouest-Est des Leucémies et Autres Maladies du Sang (2014). High level of soluble programmed cell death ligand 1 in blood impacts overall survival in aggressive diffuse large B-Cell lymphoma: results from a French multicenter clinical trial. Leukemia.

[44] Kruger S, Legenstein ML, Rösgen V, Haas M, Modest DP, Westphalen CB, Ormanns S, Kirchner T, Heinemann V, Holdenrieder S, Boeck S (2017). Serum levels of soluble programmed death protein 1 (sPD-1) and soluble programmed death ligand 1 (sPD-L1) in advanced pancreatic cancer. Oncoimmunology.

[45] Costantini A, Julie C, Dumenil C, Hélias-Rodzewicz Z, Tisserand J, Dumoulin J, Giraud V, Labrune S, Chinet T, Emile JF, Giroux LE (2018). Predictive role of plasmatic biomarkers in advanced non-small cell lung cancer treated by nivolumab. Oncoimmunology.

[46] Sanmamed MF, Perez-Gracia JL, Schalper KA, Fusco JP, Gonzalez A, Rodriguez-Ruiz ME, Oñate C, Perez G, Alfaro C, Martín-Algarra S, Andueza MP, Gurpide A, Morgado M, Wang J, Bacchiocchi A, Halaban R, Kluger H, Chen L, Sznol M, Melero I (2017). Changes in serum interleukin-8 (IL-8) levels reflect and predict response to anti-PD-1 treatment in melanoma and non-small-cell lung cancer patients. Ann Oncol.

[47] Sanmamed MF, Carranza-Rua O, Alfaro C, Oñate C, Martín-Algarra S, Perez G, Landazuri SF, Gonzalez A, Gross S, Rodriguez I, Muñoz-Calleja C, Rodríguez-Ruiz M, Sangro B, López-Picazo JM, Rizzo M, Mazzolini G, Pascual JI, Andueza MP, Perez-Gracia JL, Melero I (2014). Serum interleukin-8 reflects tumor burden and treatment response across malignancies of multiple tissue origins. Clin Cancer Res.

[48] Juliá EP, Mandó P, Rizzo MM, Cueto GR, Tsou F, Luca R, Pupareli C, Bravo AI, Astorino W, Mordoh J, Martín C, Levy EM (2019). Peripheral changes in immune cell populations and soluble mediators after anti-PD-1 therapy in non-small cell lung cancer and renal cell carcinoma patients. Cancer Immunol Immunother.

[49] Daassi D, Mahoney KM, Freeman GJ (2020). The importance of exosomal PDL1 in tumour immune evasion. Nat Rev Immunol.

