# Supplementary material for: Association between response to anti-PD-1 treatment and blood soluble PD-L1 and IL-8 changes in patients with NSCLC
Source: Discov Oncol. 2023 Mar 29;14:35. doi: 10.1007/s12672-023-00641-2 (PMC10060455; doi:10.1007/s12672-023-00641-2)
Supplement: Supplementary file 1 — Additional file1 (DOCX 12 KB) [file 12672_2023_641_MOESM1_ESM.docx]

**Supplementary Table 1 ForteBio analysis of 11E3 binding to PD-L1-His**

| Conc. (nM) | Response | KD (M) | KD Error | kon(1/Ms) | kon Error | kdis(1/s) | kdis Error |
| --- | --- | --- | --- | --- | --- | --- | --- |
| 100 | 0.0658 | 1.80E-09 | 2.35E-11 | 7.55E+05 | 6.78E+03 | 1.36E-03 | 1.30E-05 |
| 50 | 0.0553 | 1.80E-09 | 2.35E-11 | 7.55E+05 | 6.78E+03 | 1.36E-03 | 1.30E-05 |
| 25 | 0.051 | 1.80E-09 | 2.35E-11 | 7.55E+05 | 6.78E+03 | 1.36E-03 | 1.30E-05 |
| 12.5 | 0.0332 | 1.80E-09 | 2.35E-11 | 7.55E+05 | 6.78E+03 | 1.36E-03 | 1.30E-05 |
| 6.25 | 0.03 | 1.80E-09 | 2.35E-11 | 7.55E+05 | 6.78E+03 | 1.36E-03 | 1.30E-05 |
| 3.13 | 0.0212 | 1.80E-09 | 2.35E-11 | 7.55E+05 | 6.78E+03 | 1.36E-03 | 1.30E-05 |
| 1.78 | 0.0144 | 1.80E-09 | 2.35E-11 | 7.55E+05 | 6.78E+03 | 1.36E-03 | 1.30E-05 |
