# Supplementary material for: Association between response to anti-PD-1 treatment and blood soluble PD-L1 and IL-8 changes in patients with NSCLC
Source: Discov Oncol. 2023 Mar 29;14:35. doi: 10.1007/s12672-023-00641-2 (PMC10060455; doi:10.1007/s12672-023-00641-2)
Supplement: Supplementary file 2 — Additional file2 (DOCX 12 KB) [file 12672_2023_641_MOESM2_ESM.docx]

**Supplementary Table 2 ForteBio analysis of 2F1 binding to PD-L1-His**

| Conc. (nM) | Response | KD (M) | KD Error | kon(1/Ms) | kon Error | kdis(1/s) | kdis Error |
| --- | --- | --- | --- | --- | --- | --- | --- |
| 100 | 0.0869 | 6.96E-09 | 1.01E-10 | 3.15E+05 | 3.67E+03 | 2.19E-03 | 1.92E-05 |
| 50 | 0.0615 | 6.96E-09 | 1.01E-10 | 3.15E+05 | 3.67E+03 | 2.19E-03 | 1.92E-05 |
| 25 | 0.0518 | 6.96E-09 | 1.01E-10 | 3.15E+05 | 3.67E+03 | 2.19E-03 | 1.92E-05 |
| 12.5 | 0.0347 | 6.96E-09 | 1.01E-10 | 3.15E+05 | 3.67E+03 | 2.19E-03 | 1.92E-05 |
| 6.25 | 0.0216 | 6.96E-09 | 1.01E-10 | 3.15E+05 | 3.67E+03 | 2.19E-03 | 1.92E-05 |
| 3.13 | 0.0134 | 6.96E-09 | 1.01E-10 | 3.15E+05 | 3.67E+03 | 2.19E-03 | 1.92E-05 |
| 1.78 | 0.0093 | 6.96E-09 | 1.01E-10 | 3.15E+05 | 3.67E+03 | 2.19E-03 | 1.92E-05 |
