# Supplementary figures and images for: Association between response to anti-PD-1 treatment and blood soluble PD-L1 and IL-8 changes in patients with NSCLC
Source: Discov Oncol. 2023 Mar 29;14:35. doi: 10.1007/s12672-023-00641-2 (PMC10060455; doi:10.1007/s12672-023-00641-2)

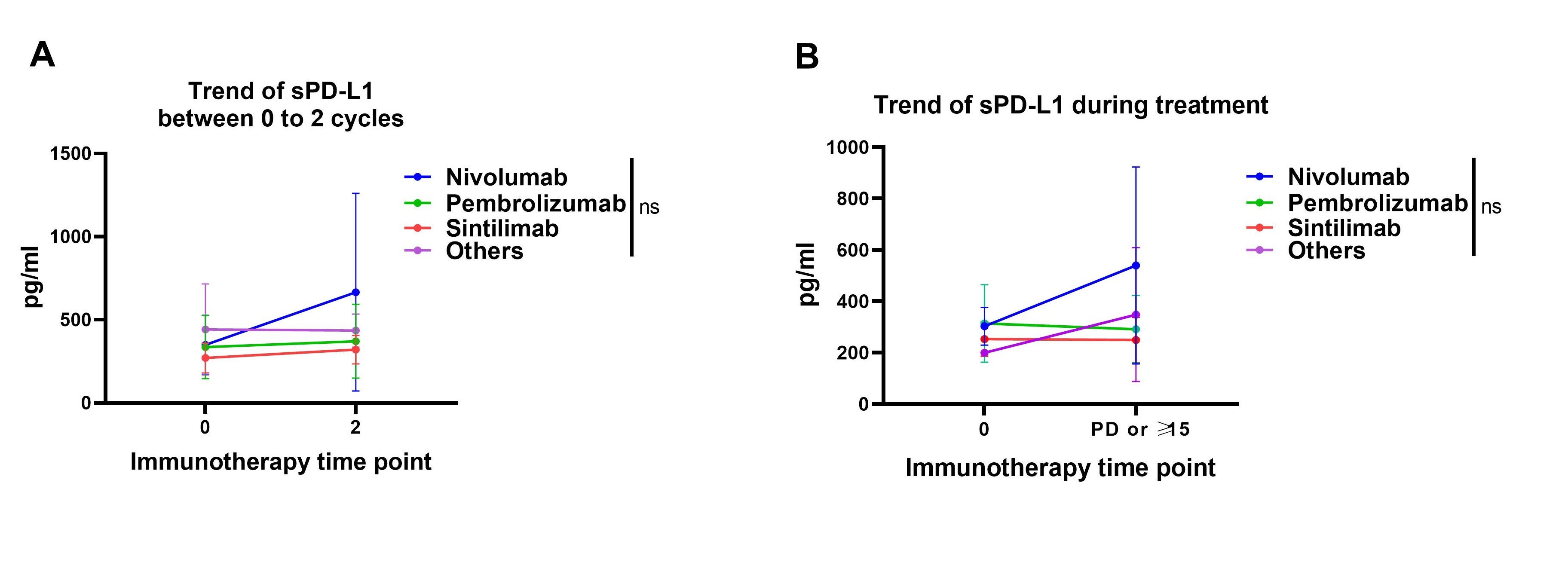

Supplement: Supplementary file 3 — Additional file 3: Figure 1 Comparison of sPD-L1 change trend among groups treated with different PD-1 antibodies during treatment. A, There was no significant difference in the change trend of sPD-L1 among different groups between 0 to 2 cycles. B, There was no significant difference in the change trend of sPD-L1 among different groups between 0 to PD or ≥15 cycles. [file 12672_2023_641_MOESM3_ESM.jpg]

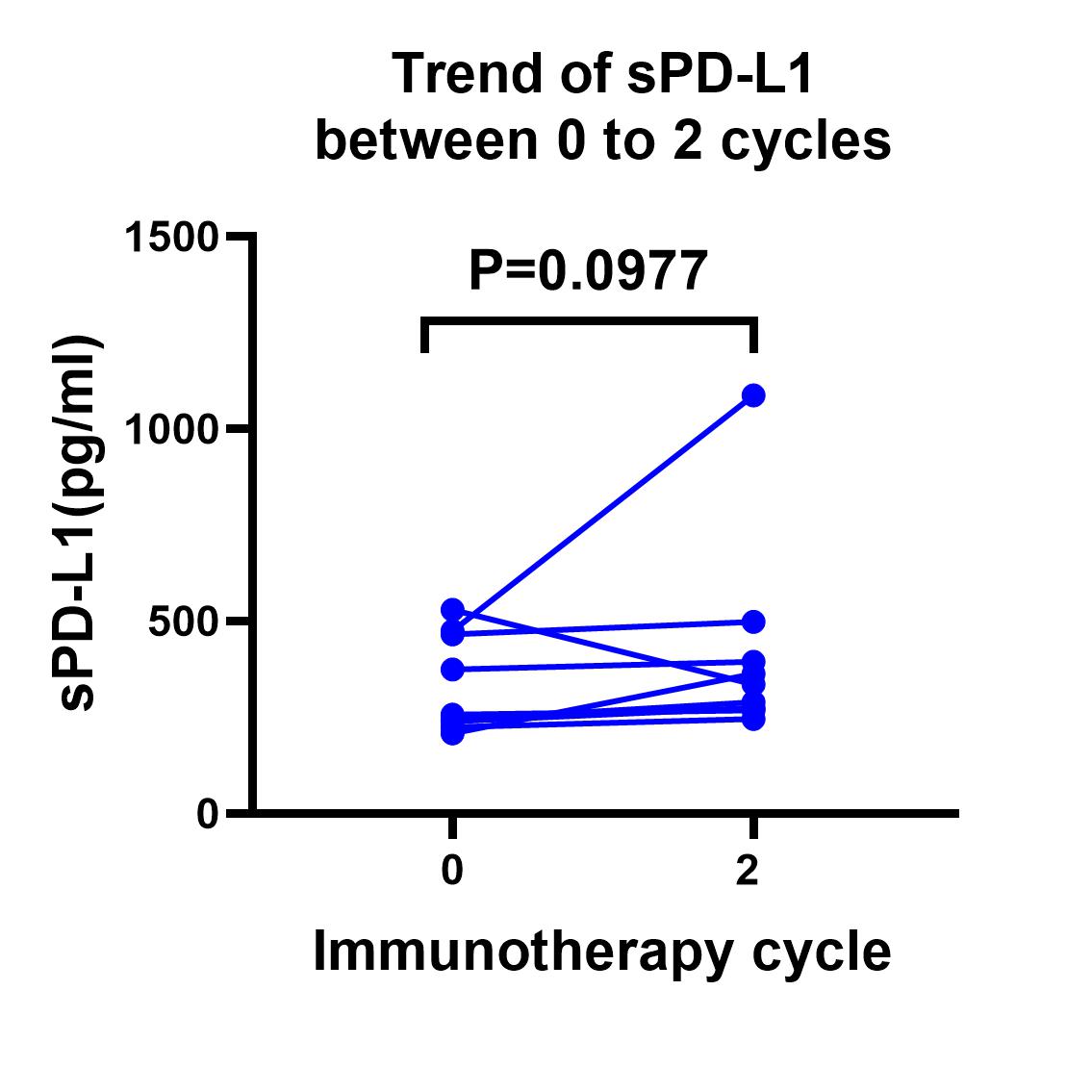

Supplement: Supplementary file 4 — Additional file 4: Figure 2 Changes in functional sPD-L1 from baseline to 2 cycles in PD patients Blood functional sPD-L1 levels rose from baseline to 2 cycles in PD patients. [file 12672_2023_641_MOESM4_ESM.jpg]

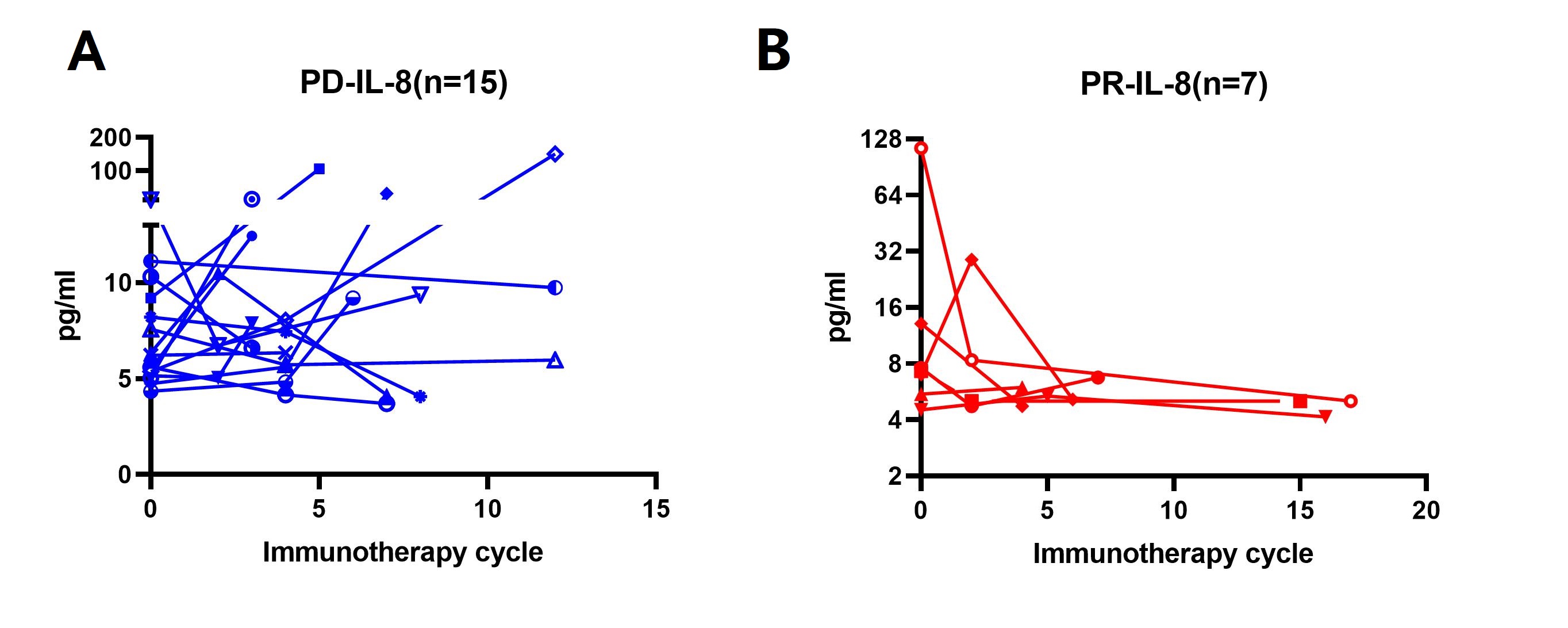

Supplement: Supplementary file 5 — Additional file 5: (JPG 247 KB): Figure 3 Dynamic changes in IL-8 during anti-PD-1 treatment. A,B, Dynamic changes in blood IL-8 in PD (n=15) and PR (n=7) patients during anti-PD-1 treatment. Each line represents a patient. [file 12672_2023_641_MOESM5_ESM.jpg]
